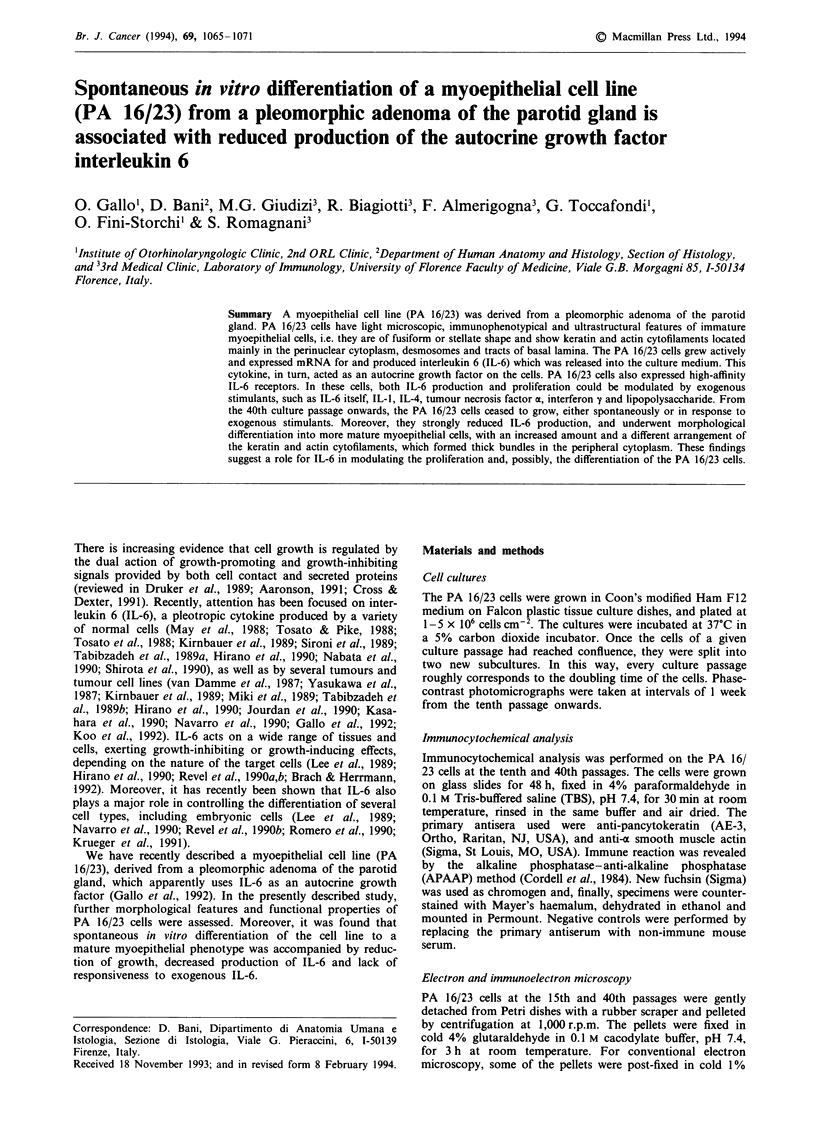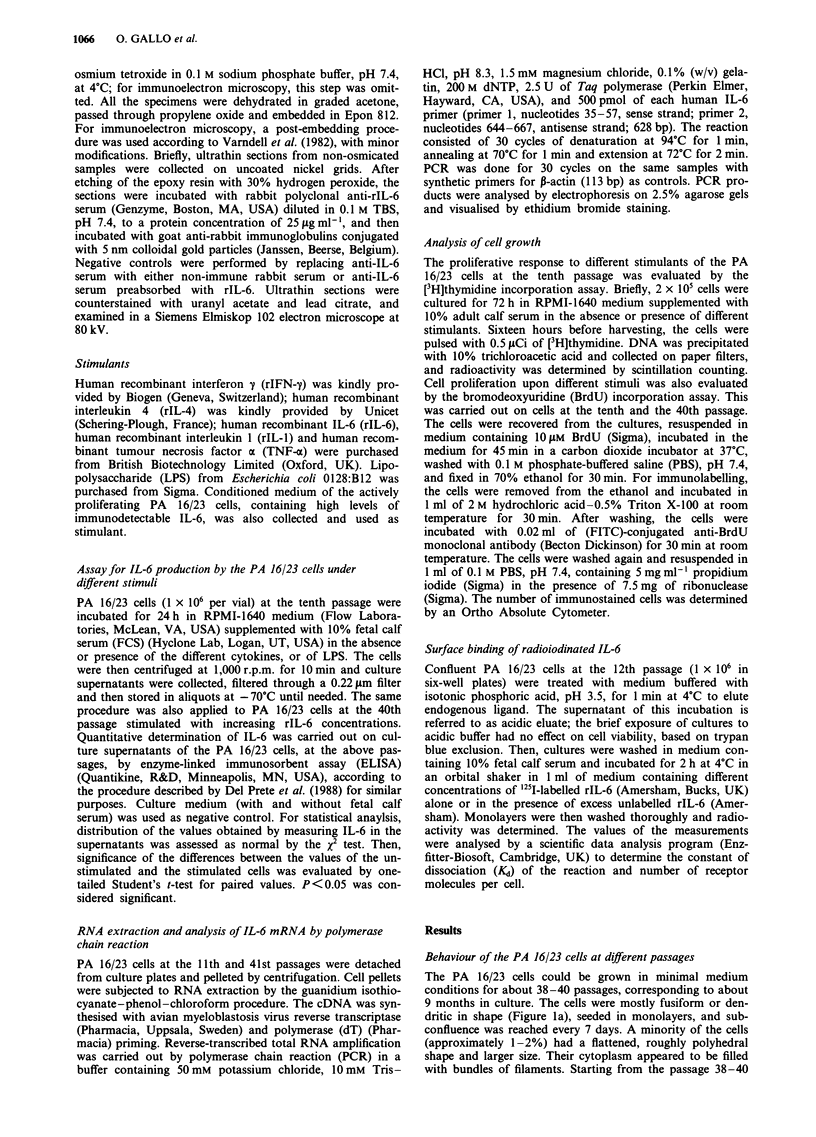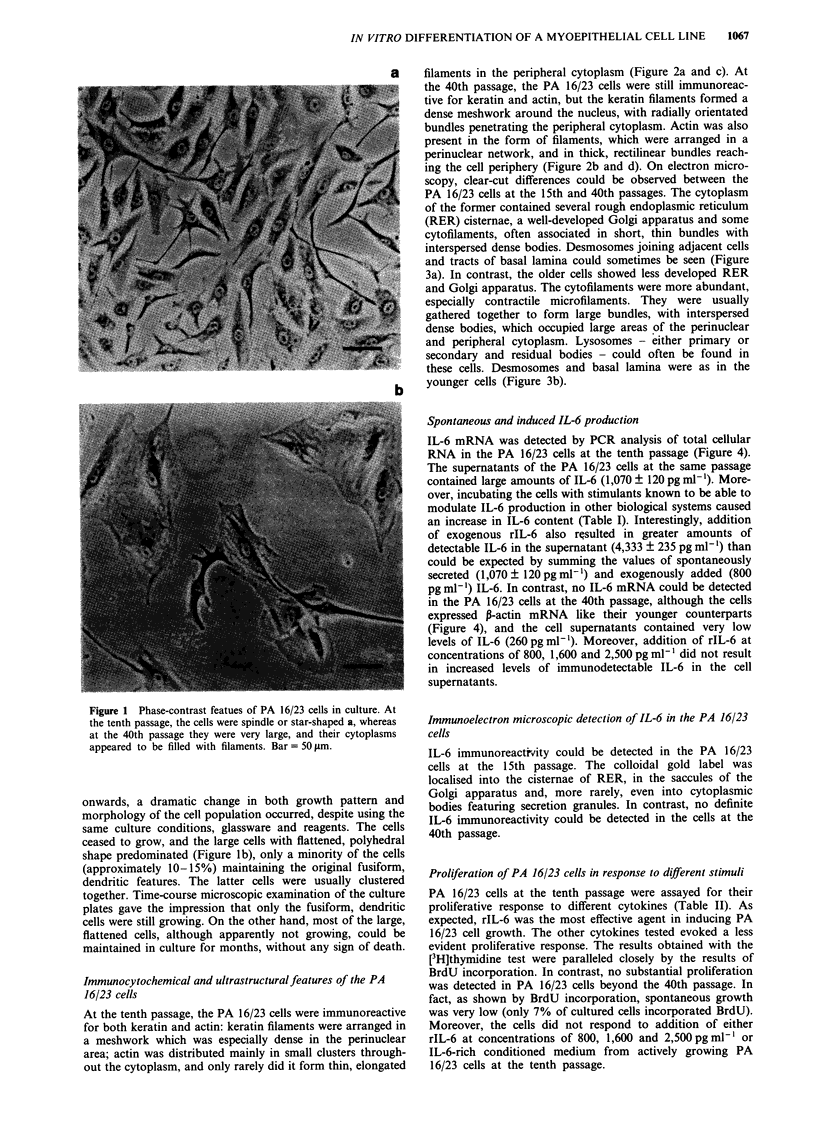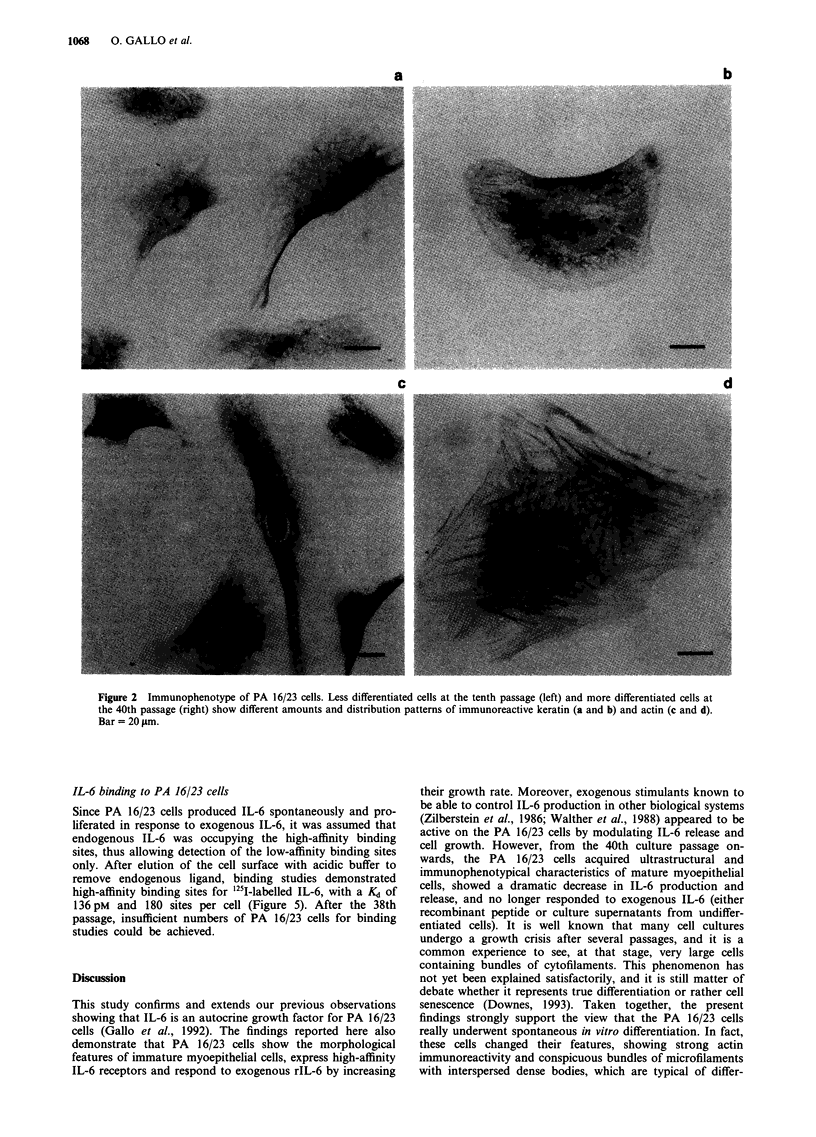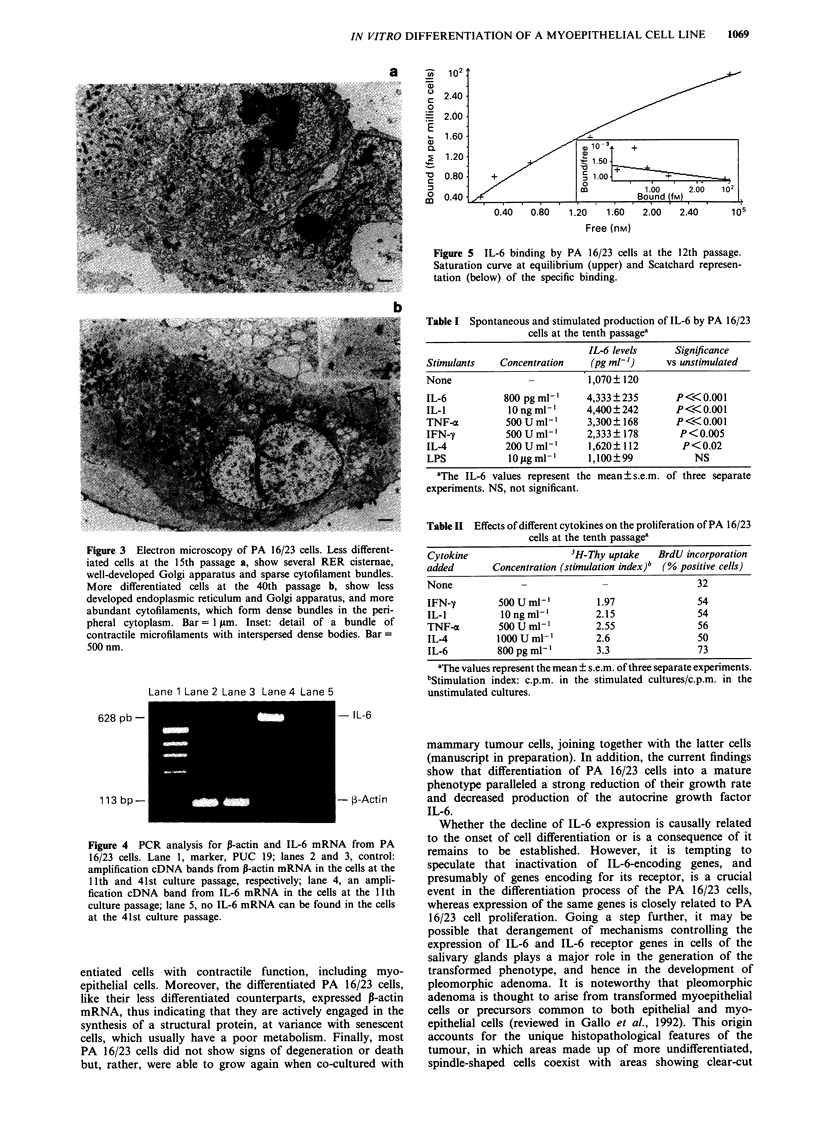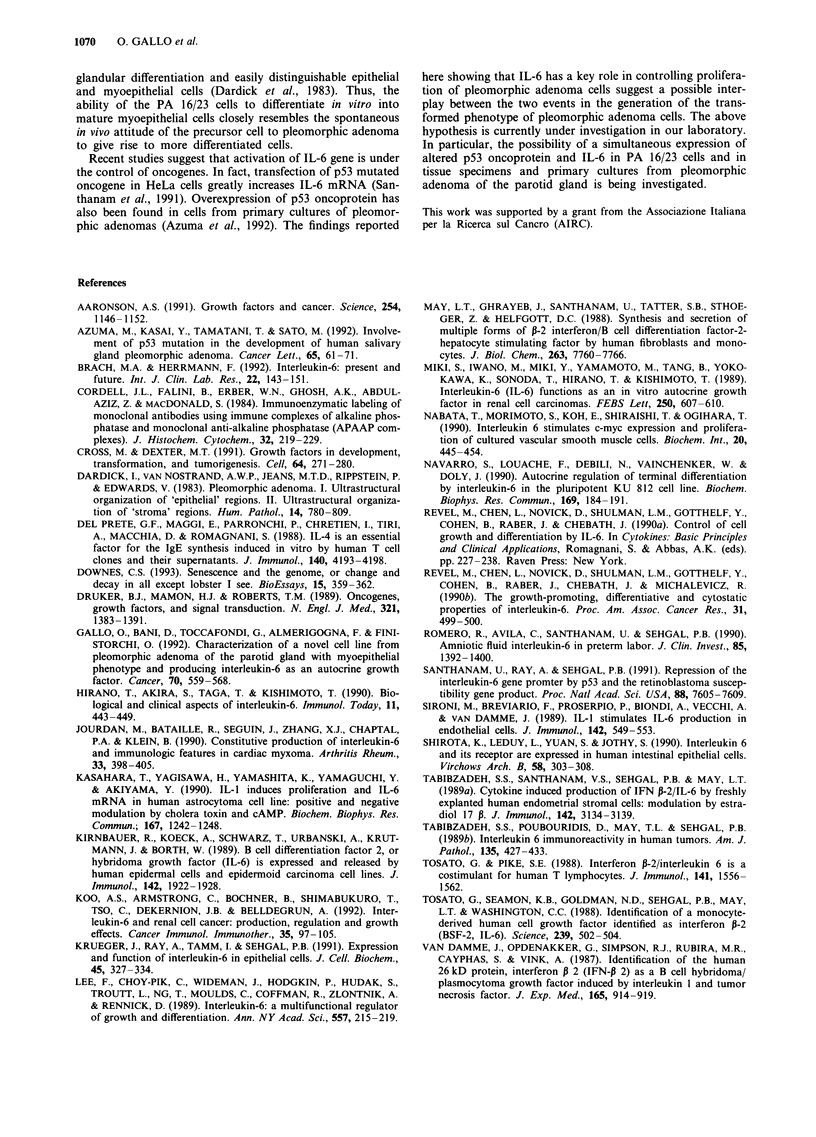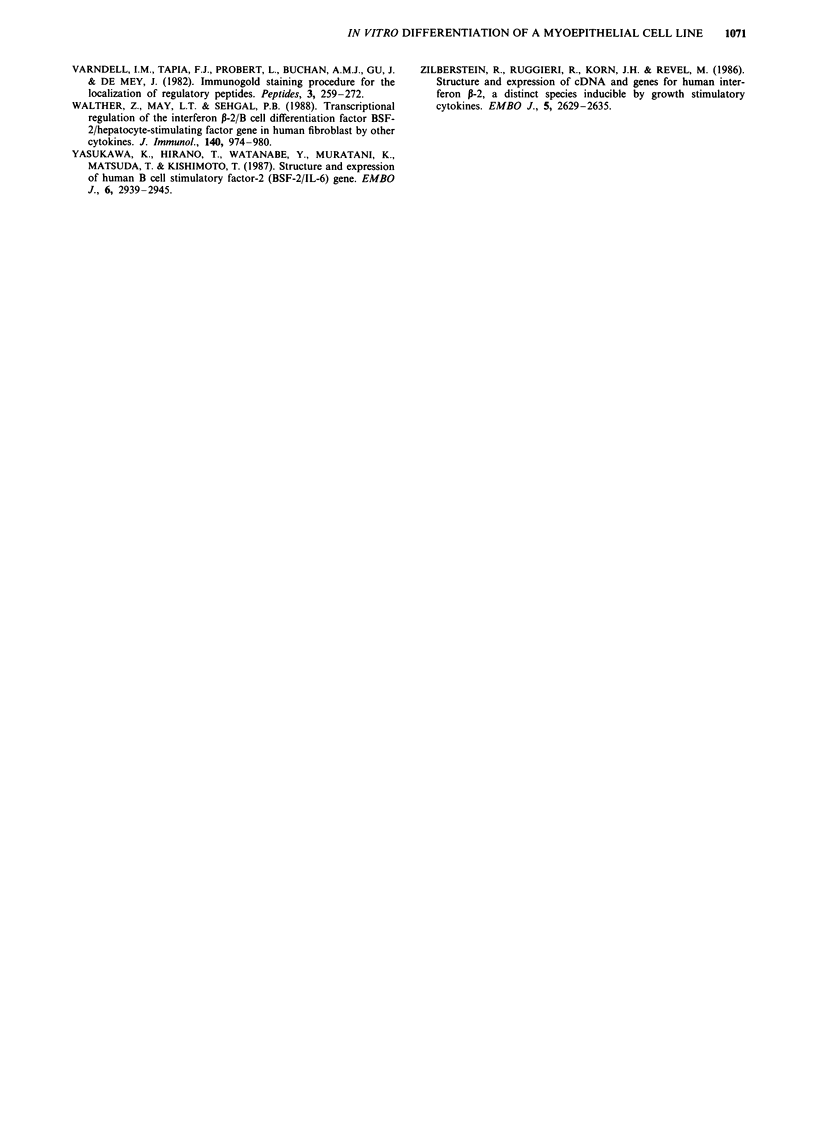# Spontaneous in vitro differentiation of a myoepithelial cell line (PA 16/23) from a pleomorphic adenoma of the parotid gland is associated with reduced production of the autocrine growth factor interleukin 6.

**DOI:** 10.1038/bjc.1994.209

**Published:** 1994-06

**Authors:** O. Gallo, D. Bani, M. G. Giudizi, R. Biagiotti, F. Almerigogna, G. Toccafondi, O. Fini-Storchi, S. Romagnani

**Affiliations:** Institute of Otorhinolaryngologic Clinic, University of Florence Faculty of Medicine, Italy.

## Abstract

**Images:**


Br. J. Cancer (1994), 69, 1065–1071

© Macmillan Press Ltd., 1994

### Spontaneous *in vitro* differentiation of a myoepithelial cell line (PA 16/23) from a pleomorphic adenoma of the parotid gland is associated with reduced production of the autocrine growth factor interleukin 6

O. Gallo[1], D. Bani[2], M.G. Giudizi[3], R. Biagiotti[3], F. Almerigogna[3], G. Toccafondi[1], O. Fini-Storchi[1] & S. Romagnani[3]

[1]*Institute of Otorhinolaryngologic Clinic, 2nd ORL Clinic,* [2]*Department of Human Anatomy and Histology, Section of Histology, and* [3]*3rd Medical Clinic, Laboratory of Immunology, University of Florence Faculty of Medicine, Viale G.B. Morgagni 85, I-50134 Florence, Italy.*

**Summary** A myoepithelial cell line (PA 16/23) was derived from a pleomorphic adenoma of the parotid gland. PA 16/23 cells have light microscopic, immunophenotypical and ultrastructural features of immature myoepithelial cells, i.e. they are of fusiform or stellate shape and show keratin and actin cytofilaments located mainly in the perinuclear cytoplasm, desmosomes and tracts of basal lamina. The PA 16/23 cells grew actively and expressed mRNA for and produced interleukin 6 (IL-6) which was released into the culture medium. This cytokine, in turn, acted as an autocrine growth factor on the cells. PA 16/23 cells also expressed high-affinity IL-6 receptors. In these cells, both IL-6 production and proliferation could be modulated by exogenous stimulants, such as IL-6 itself, IL-1, IL-4, tumour necrosis factor α, interferon γ and lipopolysaccharide. From the 40th culture passage onwards, the PA 16/23 cells ceased to grow, either spontaneously or in response to exogenous stimulants. Moreover, they strongly reduced IL-6 production, and underwent morphological differentiation into more mature myoepithelial cells, with an increased amount and a different arrangement of the keratin and actin cytofilaments, which formed thick bundles in the peripheral cytoplasm. These findings suggest a role for IL-6 in modulating the proliferation and, possibly, the differentiation of the PA 16/23 cells.

There is increasing evidence that cell growth is regulated by the dual action of growth-promoting and growth-inhibiting signals provided by both cell contact and secreted proteins (reviewed in Druker *et al.*, 1989; Aaronson, 1991; Cross & Dexter, 1991). Recently, attention has been focused on interleukin 6 (IL-6), a pleotropic cytokine produced by a variety of normal cells (May *et al.*, 1988; Tosato & Pike, 1988; Tosato *et al.*, 1988; Kirnbauer *et al.*, 1989; Sironi *et al.*, 1989; Tabibzadeh *et al.*, 1989*a*, Hirano *et al.*, 1990; Nabata *et al.*, 1990; Shirota *et al.*, 1990), as well as by several tumours and tumour cell lines (van Damme *et al.*, 1987; Yasukawa *et al.*, 1987; Kirnbauer *et al.*, 1989; Miki *et al.*, 1989; Tabibzadeh *et al.*, 1989*b*; Hirano *et al.*, 1990; Jourdan *et al.*, 1990; Kasahara *et al.*, 1990; Navarro *et al.*, 1990; Gallo *et al.*, 1992; Koo *et al.*, 1992). IL-6 acts on a wide range of tissues and cells, exerting growth-inhibiting or growth-inducing effects, depending on the nature of the target cells (Lee *et al.*, 1989; Hirano *et al.*, 1990; Revel *et al.*, 1990*a*,*b*; Brach & Herrmann, 1992). Moreover, it has recently been shown that IL-6 also plays a major role in controlling the differentiation of several cell types, including embryonic cells (Lee *et al.*, 1989; Navarro *et al.*, 1990; Revel *et al.*, 1990*b*; Romero *et al.*, 1990; Krueger *et al.*, 1991).

We have recently described a myoepithelial cell line (PA 16/23), derived from a pleomorphic adenoma of the parotid gland, which apparently uses IL-6 as an autocrine growth factor (Gallo *et al.*, 1992). In the presently described study, further morphological features and functional properties of PA 16/23 cells were assessed. Moreover, it was found that spontaneous *in vitro* differentiation of the cell line to a mature myoepithelial phenotype was accompanied by reduction of growth, decreased production of IL-6 and lack of responsiveness to exogenous IL-6.

Correspondence: D. Bani, Dipartimento di Anatomia Umana e Istologia, Sezione di Istologia, Viale G. Pieraccini, 6, I-50139 Firenze, Italy.

Received 18 November 1993; and in revised form 8 February 1994.

### Materials and methods

### Cell cultures

The PA 16/23 cells were grown in Coon's modified Ham F12 medium on Falcon plastic tissue culture dishes, and plated at $1-5 \times 10^6$ cells $cm^{-2}$. The cultures were incubated at 37°C in a 5% carbon dioxide incubator. Once the cells of a given culture passage had reached confluence, they were split into two new subcultures. In this way, every culture passage roughly corresponds to the doubling time of the cells. Phase-contrast photomicrographs were taken at intervals of 1 week from the tenth passage onwards.

### Immunocytochemical analysis

Immunocytochemical analysis was performed on the PA 16/23 cells at the tenth and 40th passages. The cells were grown on glass slides for 48 h, fixed in 4% paraformaldehyde in 0.1 M Tris-buffered saline (TBS), pH 7.4, for 30 min at room temperature, rinsed in the same buffer and air dried. The primary antisera used were anti-pancytokeratin (AE-3, Ortho, Raritan, NJ, USA), and anti-α smooth muscle actin (Sigma, St Louis, MO, USA). Immune reaction was revealed by the alkaline phosphatase–anti-alkaline phosphatase (APAAP) method (Cordell *et al.*, 1984). New fuchsin (Sigma) was used as chromogen and, finally, specimens were counterstained with Mayer's haemalum, dehydrated in ethanol and mounted in Permount. Negative controls were performed by replacing the primary antiserum with non-immune mouse serum.

### Electron and immunoelectron microscopy

PA 16/23 cells at the 15th and 40th passages were gently detached from Petri dishes with a rubber scraper and pelleted by centrifugation at 1,000 r.p.m. The pellets were fixed in cold 4% glutaraldehyde in 0.1 M cacodylate buffer, pH 7.4, for 3 h at room temperature. For conventional electron microscopy, some of the pellets were post-fixed in cold 1%

osmium tetroxide in 0.1 M sodium phosphate buffer, pH 7.4, at 4°C; for immunoelectron microscopy, this step was omitted. All the specimens were dehydrated in graded acetone, passed through propylene oxide and embedded in Epon 812. For immunoelectron microscopy, a post-embedding procedure was used according to Varndell *et al.* (1982), with minor modifications. Briefly, ultrathin sections from non-osmicated samples were collected on uncoated nickel grids. After etching of the epoxy resin with 30% hydrogen peroxide, the sections were incubated with rabbit polyclonal anti-rIL-6 serum (Genzyme, Boston, MA, USA) diluted in 0.1 M TBS, pH 7.4, to a protein concentration of 25 $\mu g\ ml^{-1}$, and then incubated with goat anti-rabbit immunoglobulins conjugated with 5 nm colloidal gold particles (Janssen, Beerse, Belgium). Negative controls were performed by replacing anti-IL-6 serum with either non-immune rabbit serum or anti-IL-6 serum preabsorbed with rIL-6. Ultrathin sections were counterstained with uranyl acetate and lead citrate, and examined in a Siemens Elmiskop 102 electron microscope at 80 kV.

### *Stimulants*

Human recombinant interferon $\gamma$ (rIFN-$\gamma$) was kindly provided by Biogen (Geneva, Switzerland); human recombinant interleukin 4 (rIL-4) was kindly provided by Unicet (Schering-Plough, France); human recombinant IL-6 (rIL-6), human recombinant interleukin 1 (rIL-1) and human recombinant tumour necrosis factor $\alpha$ (TNF-$\alpha$) were purchased from British Biotechnology Limited (Oxford, UK). Lipopolysaccharide (LPS) from *Escherichia coli* 0128:B12 was purchased from Sigma. Conditioned medium of the actively proliferating PA 16/23 cells, containing high levels of immunodetectable IL-6, was also collected and used as stimulant.

### *Assay for IL-6 production by the PA 16/23 cells under different stimuli*

PA 16/23 cells ($1 \times 10^6$ per vial) at the tenth passage were incubated for 24 h in RPMI-1640 medium (Flow Laboratories, McLean, VA, USA) supplemented with 10% fetal calf serum (FCS) (Hyclone Lab, Logan, UT, USA) in the absence or presence of the different cytokines, or of LPS. The cells were then centrifuged at 1,000 r.p.m. for 10 min and culture supernatants were collected, filtered through a 0.22 $\mu m$ filter and then stored in aliquots at −70°C until needed. The same procedure was also applied to PA 16/23 cells at the 40th passage stimulated with increasing rIL-6 concentrations. Quantitative determination of IL-6 was carried out on culture supernatants of the PA 16/23 cells, at the above passages, by enzyme-linked immunosorbent assay (ELISA) (Quantikine, R&D, Minneapolis, MN, USA), according to the procedure described by Del Prete *et al.* (1988) for similar purposes. Culture medium (with and without fetal calf serum) was used as negative control. For statistical anaylsis, distribution of the values obtained by measuring IL-6 in the supernatants was assessed as normal by the $\chi^2$ test. Then, significance of the differences between the values of the unstimulated and the stimulated cells was evaluated by one-tailed Student's *t*-test for paired values. $P < 0.05$ was considered significant.

### *RNA extraction and analysis of IL-6 mRNA by polymerase chain reaction*

PA 16/23 cells at the 11th and 41st passages were detached from culture plates and pelleted by centrifugation. Cell pellets were subjected to RNA extraction by the guanidium isothiocyanate–phenol–chloroform procedure. The cDNA was synthesised with avian myeloblastosis virus reverse transcriptase (Pharmacia, Uppsala, Sweden) and polymerase (dT) (Pharmacia) priming. Reverse-transcribed total RNA amplification was carried out by polymerase chain reaction (PCR) in a buffer containing 50 mM potassium chloride, 10 mM Tris–HCl, pH 8.3, 1.5 mM magnesium chloride, 0.1% (w/v) gelatin, 200 M dNTP, 2.5 U of *Taq* polymerase (Perkin Elmer, Hayward, CA, USA), and 500 pmol of each human IL-6 primer (primer 1, nucleotides 35–57, sense strand; primer 2, nucleotides 644–667, antisense strand; 628 bp). The reaction consisted of 30 cycles of denaturation at 94°C for 1 min, annealing at 70°C for 1 min and extension at 72°C for 2 min. PCR was done for 30 cycles on the same samples with synthetic primers for $\beta$-actin (113 bp) as controls. PCR products were analysed by electrophoresis on 2.5% agarose gels and visualised by ethidium bromide staining.

### *Analysis of cell growth*

The proliferative response to different stimulants of the PA 16/23 cells at the tenth passage was evaluated by the [$^3$H]thymidine incorporation assay. Briefly, $2 \times 10^5$ cells were cultured for 72 h in RPMI-1640 medium supplemented with 10% adult calf serum in the absence or presence of different stimulants. Sixteen hours before harvesting, the cells were pulsed with 0.5 $\mu$Ci of [$^3$H]thymidine. DNA was precipitated with 10% trichloroacetic acid and collected on paper filters, and radioactivity was determined by scintillation counting. Cell proliferation upon different stimuli was also evaluated by the bromodeoxyuridine (BrdU) incorporation assay. This was carried out on cells at the tenth and the 40th passage. The cells were recovered from the cultures, resuspended in medium containing 10 $\mu$M BrdU (Sigma), incubated in the medium for 45 min in a carbon dioxide incubator at 37°C, washed with 0.1 M phosphate-buffered saline (PBS), pH 7.4, and fixed in 70% ethanol for 30 min. For immunolabelling, the cells were removed from the ethanol and incubated in 1 ml of 2 M hydrochloric acid–0.5% Triton X-100 at room temperature for 30 min. After washing, the cells were incubated with 0.02 ml of (FITC)-conjugated anti-BrdU monoclonal antibody (Becton Dickinson) for 30 min at room temperature. The cells were washed again and resuspended in 1 ml of 0.1 M PBS, pH 7.4, containing 5 $mg\ ml^{-1}$ propidium iodide (Sigma) in the presence of 7.5 mg of ribonuclease (Sigma). The number of immunostained cells was determined by an Ortho Absolute Cytometer.

### *Surface binding of radioiodinated IL-6*

Confluent PA 16/23 cells at the 12th passage ($1 \times 10^6$ in six-well plates) were treated with medium buffered with isotonic phosphoric acid, pH 3.5, for 1 min at 4°C to elute endogenous ligand. The supernatant of this incubation is referred to as acidic eluate; the brief exposure of cultures to acidic buffer had no effect on cell viability, based on trypan blue exclusion. Then, cultures were washed in medium containing 10% fetal calf serum and incubated for 2 h at 4°C in an orbital shaker in 1 ml of medium containing different concentrations of $^{125}$I-labelled rIL-6 (Amersham, Bucks, UK) alone or in the presence of excess unlabelled rIL-6 (Amersham). Monolayers were then washed thoroughly and radioactivity was determined. The values of the measurements were analysed by a scientific data analysis program (Enzfitter-Biosoft, Cambridge, UK) to determine the constant of dissociation ($K_d$) of the reaction and number of receptor molecules per cell.

### Results

### *Behaviour of the PA 16/23 cells at different passages*

The PA 16/23 cells could be grown in minimal medium conditions for about 38–40 passages, corresponding to about 9 months in culture. The cells were mostly fusiform or dendritic in shape (Figure 1a), seeded in monolayers, and subconfluence was reached every 7 days. A minority of the cells (approximately 1–2%) had a flattened, roughly polyhedral shape and larger size. Their cytoplasm appeared to be filled with bundles of filaments. Starting from the passage 38–40

**Figure 1** Phase-contrast featues of PA 16/23 cells in culture. At the tenth passage, the cells were spindle or star-shaped **a**, whereas at the 40th passage they were very large, and their cytoplasms appeared to be filled with filaments. Bar = 50 μm.

onwards, a dramatic change in both growth pattern and morphology of the cell population occurred, despite using the same culture conditions, glassware and reagents. The cells ceased to grow, and the large cells with flattened, polyhedral shape predominated (Figure 1b), only a minority of the cells (approximately 10–15%) maintaining the original fusiform, dendritic features. The latter cells were usually clustered together. Time-course microscopic examination of the culture plates gave the impression that only the fusiform, dendritic cells were still growing. On the other hand, most of the large, flattened cells, although apparently not growing, could be maintained in culture for months, without any sign of death.

*Immunocytochemical and ultrastructural features of the PA 16/23 cells*

At the tenth passage, the PA 16/23 cells were immunoreactive for both keratin and actin: keratin filaments were arranged in a meshwork which was especially dense in the perinuclear area; actin was distributed mainly in small clusters throughout the cytoplasm, and only rarely did it form thin, elongated filaments in the peripheral cytoplasm (Figure 2a and c). At the 40th passage, the PA 16/23 cells were still immunoreactive for keratin and actin, but the keratin filaments formed a dense meshwork around the nucleus, with radially orientated bundles penetrating the peripheral cytoplasm. Actin was also present in the form of filaments, which were arranged in a perinuclear network, and in thick, rectilinear bundles reaching the cell periphery (Figure 2b and d). On electron microscopy, clear-cut differences could be observed between the PA 16/23 cells at the 15th and 40th passages. The cytoplasm of the former contained several rough endoplasmic reticulum (RER) cisternae, a well-developed Golgi apparatus and some cytofilaments, often associated in short, thin bundles with interspersed dense bodies. Desmosomes joining adjacent cells and tracts of basal lamina could sometimes be seen (Figure 3a). In contrast, the older cells showed less developed RER and Golgi apparatus. The cytofilaments were more abundant, especially contractile microfilaments. They were usually gathered together to form large bundles, with interspersed dense bodies, which occupied large areas of the perinuclear and peripheral cytoplasm. Lysosomes – either primary or secondary and residual bodies – could often be found in these cells. Desmosomes and basal lamina were as in the younger cells (Figure 3b).

*Spontaneous and induced IL-6 production*

IL-6 mRNA was detected by PCR analysis of total cellular RNA in the PA 16/23 cells at the tenth passage (Figure 4). The supernatants of the PA 16/23 cells at the same passage contained large amounts of IL-6 (1,070 ± 120 pg ml$^{-1}$). Moreover, incubating the cells with stimulants known to be able to modulate IL-6 production in other biological systems caused an increase in IL-6 content (Table I). Interestingly, addition of exogenous rIL-6 also resulted in greater amounts of detectable IL-6 in the supernatant (4,333 ± 235 pg ml$^{-1}$) than could be expected by summing the values of spontaneously secreted (1,070 ± 120 pg ml$^{-1}$) and exogenously added (800 pg ml$^{-1}$) IL-6. In contrast, no IL-6 mRNA could be detected in the PA 16/23 cells at the 40th passage, although the cells expressed β-actin mRNA like their younger counterparts (Figure 4), and the cell supernatants contained very low levels of IL-6 (260 pg ml$^{-1}$). Moreover, addition of rIL-6 at concentrations of 800, 1,600 and 2,500 pg ml$^{-1}$ did not result in increased levels of immunodetectable IL-6 in the cell supernatants.

*Immunoelectron microscopic detection of IL-6 in the PA 16/23 cells*

IL-6 immunoreactivity could be detected in the PA 16/23 cells at the 15th passage. The colloidal gold label was localised into the cisternae of RER, in the saccules of the Golgi apparatus and, more rarely, even into cytoplasmic bodies featuring secretion granules. In contrast, no definite IL-6 immunoreactivity could be detected in the cells at the 40th passage.

*Proliferation of PA 16/23 cells in response to different stimuli*

PA 16/23 cells at the tenth passage were assayed for their proliferative response to different cytokines (Table II). As expected, rIL-6 was the most effective agent in inducing PA 16/23 cell growth. The other cytokines tested evoked a less evident proliferative response. The results obtained with the [$^3$H]thymidine test were paralleled closely by the results of BrdU incorporation. In contrast, no substantial proliferation was detected in PA 16/23 cells beyond the 40th passage. In fact, as shown by BrdU incorporation, spontaneous growth was very low (only 7% of cultured cells incorporated BrdU). Moreover, the cells did not respond to addition of either rIL-6 at concentrations of 800, 1,600 and 2,500 pg ml$^{-1}$ or IL-6-rich conditioned medium from actively growing PA 16/23 cells at the tenth passage.

**Figure 2** Immunophenotype of PA 16/23 cells. Less differentiated cells at the tenth passage (left) and more differentiated cells at the 40th passage (right) show different amounts and distribution patterns of immunoreactive keratin (**a** and **b**) and actin (**c** and **d**). Bar = 20 μm.

*IL-6 binding to PA 16/23 cells*

Since PA 16/23 cells produced IL-6 spontaneously and proliferated in response to exogenous IL-6, it was assumed that endogenous IL-6 was occupying the high-affinity binding sites, thus allowing detection of the low-affinity binding sites only. After elution of the cell surface with acidic buffer to remove endogenous ligand, binding studies demonstrated high-affinity binding sites for $^{125}$I-labelled IL-6, with a $K_d$ of 136 pM and 180 sites per cell (Figure 5). After the 38th passage, insufficient numbers of PA 16/23 cells for binding studies could be achieved.

### Discussion

This study confirms and extends our previous observations showing that IL-6 is an autocrine growth factor for PA 16/23 cells (Gallo *et al.*, 1992). The findings reported here also demonstrate that PA 16/23 cells show the morphological features of immature myoepithelial cells, express high-affinity IL-6 receptors and respond to exogenous rIL-6 by increasing their growth rate. Moreover, exogenous stimulants known to be able to control IL-6 production in other biological systems (Zilberstein *et al.*, 1986; Walther *et al.*, 1988) appeared to be active on the PA 16/23 cells by modulating IL-6 release and cell growth. However, from the 40th culture passage onwards, the PA 16/23 cells acquired ultrastructural and immunophenotypical characteristics of mature myoepithelial cells, showed a dramatic decrease in IL-6 production and release, and no longer responded to exogenous IL-6 (either recombinant peptide or culture supernatants from undifferentiated cells). It is well known that many cell cultures undergo a growth crisis after several passages, and it is a common experience to see, at that stage, very large cells containing bundles of cytofilaments. This phenomenon has not yet been explained satisfactorily, and it is still matter of debate whether it represents true differentiation or rather cell senescence (Downes, 1993). Taken together, the present findings strongly support the view that the PA 16/23 cells really underwent spontaneous *in vitro* differentiation. In fact, these cells changed their features, showing strong actin immunoreactivity and conspicuous bundles of microfilaments with interspersed dense bodies, which are typical of differ-

**Figure 3** Electron microscopy of PA 16/23 cells. Less differentiated cells at the 15th passage **a**, show several RER cisternae, well-developed Golgi apparatus and sparse cytofilament bundles. More differentiated cells at the 40th passage **b**, show less developed endoplasmic reticulum and Golgi apparatus, and more abundant cytofilaments, which form dense bundles in the peripheral cytoplasm. Bar = 1 µm. Inset: detail of a bundle of contractile microfilaments with interspersed dense bodies. Bar = 500 nm.

**Figure 4** PCR analysis for β-actin and IL-6 mRNA from PA 16/23 cells. Lane 1, marker, PUC 19; lanes 2 and 3, control: amplification cDNA bands from β-actin mRNA in the cells at the 11th and 41st culture passage, respectively; lane 4, an amplification cDNA band from IL-6 mRNA in the cells at the 11th culture passage; lane 5, no IL-6 mRNA can be found in the cells at the 41st culture passage.

**Figure 5** IL-6 binding by PA 16/23 cells at the 12th passage. Saturation curve at equilibrium (upper) and Scatchard representation (below) of the specific binding.

**Table I** Spontaneous and stimulated production of IL-6 by PA 16/23 cells at the tenth passage[a]

| *Stimulants* | *Concentration* | *IL-6 levels* *($pg\ ml^{-1}$)* | *Significance* vs *unstimulated* |
|---|---|---|---|
| None | – | 1,070 ± 120 | |
| IL-6 | 800 pg ml$^{-1}$ | 4,333 ± 235 | $P \ll 0.001$ |
| IL-1 | 10 ng ml$^{-1}$ | 4,400 ± 242 | $P \ll 0.001$ |
| TNF-α | 500 U ml$^{-1}$ | 3,300 ± 168 | $P \ll 0.001$ |
| IFN-γ | 500 U ml$^{-1}$ | 2,333 ± 178 | $P < 0.005$ |
| IL-4 | 200 U ml$^{-1}$ | 1,620 ± 112 | $P < 0.02$ |
| LPS | 10 µg ml$^{-1}$ | 1,100 ± 99 | NS |

[a]The IL-6 values represent the mean ± s.e.m. of three separate experiments. NS, not significant.

**Table II** Effects of different cytokines on the proliferation of PA 16/23 cells at the tenth passage[a]

| *Cytokine added* | *Concentration* | *$^3$H-Thy uptake (stimulation index)*[b] | *BrdU incorporation (% positive cells)* |
|---|---|---|---|
| None | – | – | 32 |
| IFN-γ | 500 U ml$^{-1}$ | 1.97 | 54 |
| IL-1 | 10 ng ml$^{-1}$ | 2.15 | 54 |
| TNF-α | 500 U ml$^{-1}$ | 2.55 | 56 |
| IL-4 | 1000 U ml$^{-1}$ | 2.6 | 50 |
| IL-6 | 800 pg ml$^{-1}$ | 3.3 | 73 |

[a]The values represent the mean ± s.e.m. of three separate experiments.
[b]Stimulation index: c.p.m. in the stimulated cultures/c.p.m. in the unstimulated cultures.

entiated cells with contractile function, including myoepithelial cells. Moreover, the differentiated PA 16/23 cells, like their less differentiated counterparts, expressed β-actin mRNA, thus indicating that they are actively engaged in the synthesis of a structural protein, at variance with senescent cells, which usually have a poor metabolism. Finally, most PA 16/23 cells did not show signs of degeneration or death but, rather, were able to grow again when co-cultured with mammary tumour cells, joining together with the latter cells (manuscript in preparation). In addition, the current findings show that differentiation of PA 16/23 cells into a mature phenotype paralleled a strong reduction of their growth rate and decreased production of the autocrine growth factor IL-6.

Whether the decline of IL-6 expression is causally related to the onset of cell differentiation or is a consequence of it remains to be established. However, it is tempting to speculate that inactivation of IL-6-encoding genes, and presumably of genes encoding for its receptor, is a crucial event in the differentiation process of the PA 16/23 cells, whereas expression of the same genes is closely related to PA 16/23 cell proliferation. Going a step further, it may be possible that derangement of mechanisms controlling the expression of IL-6 and IL-6 receptor genes in cells of the salivary glands plays a major role in the generation of the transformed phenotype, and hence in the development of pleomorphic adenoma. It is noteworthy that pleomorphic adenoma is thought to arise from transformed myoepithelial cells or precursors common to both epithelial and myoepithelial cells (reviewed in Gallo *et al.*, 1992). This origin accounts for the unique histopathological features of the tumour, in which areas made up of more undifferentiated, spindle-shaped cells coexist with areas showing clear-cut glandular differentiation and easily distinguishable epithelial and myoepithelial cells (Dardick et al., 1983). Thus, the ability of the PA 16/23 cells to differentiate *in vitro* into mature myoepithelial cells closely resembles the spontaneous *in vivo* attitude of the precursor cell to pleomorphic adenoma to give rise to more differentiated cells.

Recent studies suggest that activation of IL-6 gene is under the control of oncogenes. In fact, transfection of p53 mutated oncogene in HeLa cells greatly increases IL-6 mRNA (Santhanam *et al.*, 1991). Overexpression of p53 oncoprotein has also been found in cells from primary cultures of pleomorphic adenomas (Azuma *et al.*, 1992). The findings reported here showing that IL-6 has a key role in controlling proliferation of pleomorphic adenoma cells suggest a possible interplay between the two events in the generation of the transformed phenotype of pleomorphic adenoma cells. The above hypothesis is currently under investigation in our laboratory. In particular, the possibility of a simultaneous expression of altered p53 oncoprotein and IL-6 in PA 16/23 cells and in tissue specimens and primary cultures from pleomorphic adenoma of the parotid gland is being investigated.

This work was supported by a grant from the Associazione Italiana per la Ricerca sul Cancro (AIRC).

### References

AARONSON, A.S. (1991). Growth factors and cancer. *Science*, **254**, 1146–1152.

AZUMA, M., KASAI, Y., TAMATANI, T. & SATO, M. (1992). Involvement of p53 mutation in the development of human salivary gland pleomorphic adenoma. *Cancer Lett.*, **65**, 61–71.

BRACH, M.A. & HERRMANN, F. (1992). Interleukin-6: present and future. *Int. J. Clin. Lab. Res.*, **22**, 143–151.

CORDELL, J.L., FALINI, B., ERBER, W.N., GHOSH, A.K., ABDUL-AZIZ, Z. & MACDONALD, S. (1984). Immunoenzymatic labeling of monoclonal antibodies using immune complexes of alkaline phosphatase and monoclonal anti-alkaline phosphatase (APAAP complexes). *J. Histochem. Cytochem.*, **32**, 219–229.

CROSS, M. & DEXTER, M.T. (1991). Growth factors in development, transformation, and tumorigenesis. *Cell*, **64**, 271–280.

DARDICK, I., VAN NOSTRAND, A.W.P., JEANS, M.T.D., RIPPSTEIN, P. & EDWARDS, V. (1983). Pleomorphic adenoma. I. Ultrastructural organization of 'epithelial' regions. II. Ultrastructural organization of 'stroma' regions. *Hum. Pathol.*, **14**, 780–809.

DEL PRETE, G.F., MAGGI, E., PARRONCHI, P., CHRETIEN, I., TIRI, A., MACCHIA, D. & ROMAGNANI, S. (1988). IL-4 is an essential factor for the IgE synthesis induced in vitro by human T cell clones and their supernatants. *J. Immunol.*, **140**, 4193–4198.

DOWNES, C.S. (1993). Senescence and the genome, or change and decay in all except lobster I see. *BioEssays*, **15**, 359–362.

DRUKER, B.J., MAMON, H.J. & ROBERTS, T.M. (1989). Oncogenes, growth factors, and signal transduction. *N. Engl. J. Med.*, **321**, 1383–1391.

GALLO, O., BANI, D., TOCCAFONDI, G., ALMERIGOGNA, F. & FINI-STORCHI, O. (1992). Characterization of a novel cell line from pleomorphic adenoma of the parotid gland with myoepithelial phenotype and producing interleukin-6 as an autocrine growth factor. *Cancer*, **70**, 559–568.

HIRANO, T., AKIRA, S., TAGA, T. & KISHIMOTO, T. (1990). Biological and clinical aspects of interleukin-6. *Immunol. Today*, **11**, 443–449.

JOURDAN, M., BATAILLE, R., SEGUIN, J., ZHANG, X.J., CHAPTAL, P.A. & KLEIN, B. (1990). Constitutive production of interleukin-6 and immunologic features in cardiac myxoma. *Arthritis Rheum.*, **33**, 398–405.

KASAHARA, T., YAGISAWA, H., YAMASHITA, K., YAMAGUCHI, Y. & AKIYAMA, Y. (1990). IL-1 induces proliferation and IL-6 mRNA in human astrocytoma cell line: positive and negative modulation by cholera toxin and cAMP. *Biochem. Biophys. Res. Commun.*; **167**, 1242–1248.

KIRNBAUER, R., KOECK, A., SCHWARZ, T., URBANSKI, A., KRUTMANN, J. & BORTH, W. (1989). B cell differentiation factor 2, or hybridoma growth factor (IL-6) is expressed and released by human epidermal cells and epidermoid carcinoma cell lines. *J. Immunol.*, **142**, 1922–1928.

KOO, A.S., ARMSTRONG, C., BOCHNER, B., SHIMABUKURO, T., TSO, C., DEKERNION, J.B. & BELLDEGRUN, A. (1992). Interleukin-6 and renal cell cancer: production, regulation and growth effects. *Cancer Immunol. Immunother.*, **35**, 97–105.

KRUEGER, J., RAY, A., TAMM, I. & SEHGAL, P.B. (1991). Expression and function of interleukin-6 in epithelial cells. *J. Cell. Biochem.*, **45**, 327–334.

LEE, F., CHOY-PIK, C., WIDEMAN, J., HODGKIN, P., HUDAK, S., TROUTT, L., NG, T., MOULDS, C., COFFMAN, R., ZLONTNIK, A. & RENNICK, D. (1989). Interleukin-6: a multifunctional regulator of growth and differentiation. *Ann. NY Acad. Sci.*, **557**, 215–219.

MAY, L.T., GHRAYEB, J., SANTHANAM, U., TATTER, S.B., STHOEGER, Z. & HELFGOTT, D.C. (1988). Synthesis and secretion of multiple forms of β-2 interferon/B cell differentiation factor-2-hepatocyte stimulating factor by human fibroblasts and monocytes. *J. Biol. Chem.*, **263**, 7760–7766.

MIKI, S., IWANO, M., MIKI, Y., YAMAMOTO, M., TANG, B., YOKOKAWA, K., SONODA, T., HIRANO, T. & KISHIMOTO, T. (1989). Interleukin-6 (IL-6) functions as an in vitro autocrine growth factor in renal cell carcinomas. *FEBS Lett*, **250**, 607–610.

NABATA, T., MORIMOTO, S., KOH, E., SHIRAISHI, T. & OGIHARA, T. (1990). Interleukin 6 stimulates c-myc expression and proliferation of cultured vascular smooth muscle cells. *Biochem. Int.*, **20**, 445–454.

NAVARRO, S., LOUACHE, F., DEBILI, N., VAINCHENKER, W. & DOLY, J. (1990). Autocrine regulation of terminal differentiation by interleukin-6 in the pluripotent KU 812 cell line. *Biochem. Biophys. Res. Commun.*, **169**, 184–191.

REVEL, M., CHEN, L., NOVICK, D., SHULMAN, L.M., GOTTHELF, Y., COHEN, B., RABER, J. & CHEBATH, J. (1990*a*). Control of cell growth and differentiation by IL-6. In *Cytokines: Basic Principles and Clinical Applications*, Romagnani, S. & Abbas, A.K. (eds). pp. 227–238. Raven Press: New York.

REVEL, M., CHEN, L., NOVICK, D., SHULMAN, L.M., GOTTHELF, Y., COHEN, B., RABER, J., CHEBATH, J. & MICHALEVICZ, R. (1990*b*). The growth-promoting, differentiative and cytostatic properties of interleukin-6. *Proc. Am. Assoc. Cancer Res.*, **31**, 499–500.

ROMERO, R., AVILA, C., SANTHANAM, U. & SEHGAL, P.B. (1990). Amniotic fluid interleukin-6 in preterm labor. *J. Clin. Invest.*, **85**, 1392–1400.

SANTHANAM, U., RAY, A. & SEHGAL, P.B. (1991). Repression of the interleukin-6 gene promter by p53 and the retinoblastoma susceptibility gene product. *Proc. Natl Acad. Sci. USA*, **88**, 7605–7609.

SIRONI, M., BREVIARIO, F., PROSERPIO, P., BIONDI, A., VECCHI, A. & VAN DAMME, J. (1989). IL-1 stimulates IL-6 production in endothelial cells. *J. Immunol.*, **142**, 549–553.

SHIROTA, K., LEDUY, L., YUAN, S. & JOTHY, S. (1990). Interleukin 6 and its receptor are expressed in human intestinal epithelial cells. *Virchows Arch. B*, **58**, 303–308.

TABIBZADEH, S.S., SANTHANAM, V.S., SEHGAL, P.B. & MAY, L.T. (1989*a*). Cytokine induced production of IFN β-2/IL-6 by freshly explanted human endometrial stromal cells: modulation by estradiol 17 β. *J. Immunol.*, **142**, 3134–3139.

TABIBZADEH, S.S., POUBOURIDIS, D., MAY, T.L. & SEHGAL, P.B. (1989*b*). Interleukin 6 immunoreactivity in human tumors. *Am. J. Pathol.*, **135**, 427–433.

TOSATO, G. & PIKE, S.E. (1988). Interferon β-2/interleukin 6 is a costimulant for human T lymphocytes. *J. Immunol.*, **141**, 1556–1562.

TOSATO, G., SEAMON, K.B., GOLDMAN, N.D., SEHGAL, P.B., MAY, L.T. & WASHINGTON, C.C. (1988). Identification of a monocyte-derived human cell growth factor identified as interferon β-2 (BSF-2, IL-6). *Science*, **239**, 502–504.

VAN DAMME, J., OPDENAKKER, G., SIMPSON, R.J., RUBIRA, M.R., CAYPHAS, S. & VINK, A. (1987). Identification of the human 26 kD protein, interferon β 2 (IFN-β 2) as a B cell hybridoma/plasmocytoma growth factor induced by interleukin 1 and tumor necrosis factor. *J. Exp. Med.*, **165**, 914–919.

VARNDELL, I.M., TAPIA, F.J., PROBERT, L., BUCHAN, A.M.J., GU, J. & DE MEY, J. (1982). Immunogold staining procedure for the localization of regulatory peptides. *Peptides*, **3**, 259–272.

WALTHER, Z., MAY, L.T. & SEHGAL, P.B. (1988). Transcriptional regulation of the interferon β-2/B cell differentiation factor BSF-2/hepatocyte-stimulating factor gene in human fibroblast by other cytokines. *J. Immunol.*, **140**, 974–980.

YASUKAWA, K., HIRANO, T., WATANABE, Y., MURATANI, K., MATSUDA, T. & KISHIMOTO, T. (1987). Structure and expression of human B cell stimulatory factor-2 (BSF-2/IL-6) gene. *EMBO J.*, **6**, 2939–2945.

ZILBERSTEIN, R., RUGGIERI, R., KORN, J.H. & REVEL, M. (1986). Structure and expression of cDNA and genes for human interferon β-2, a distinct species inducible by growth stimulatory cytokines. *EMBO J.*, **5**, 2629–2635.